# Access to technology, internet usage, and online health information-seeking behaviors in a racially diverse, lower-income population

**DOI:** 10.3389/fpubh.2024.1328544

**Published:** 2024-02-21

**Authors:** Omolola E. Adepoju, Maya Singh, Mary Tipton, Gerard Peperone, Marlen Trujillo, Chinedum Ojinnaka

**Affiliations:** ^1^Department of Health Systems and Population Health Sciences, University of Houston, Houston, TX, United States; ^2^Humana Integrated Health System Sciences Institutes, University of Houston, Houston, TX, United States; ^3^Spring Branch Community Health Center, Houston, TX, United States; ^4^College of Health Solutions, Arizona State University, Tempe, AZ, United States

**Keywords:** internet use, perception, trust, technology, health disparities

## Abstract

**Background:**

This study examined access to technology, internet usage, and online health information-seeking behaviors, in a racially diverse, lower-income population.

**Methods:**

Data were obtained via a cross-sectional survey of low-income communities in Houston, Los Angeles, and New York between April and August 2023. Binary responses to the following online health information-seeking behaviors, internet and technology access, were examined: using the internet to (i) understand a medical diagnosis, (ii) fill a prescription, (iii) schedule a healthcare appointment, (iv) email communication with a healthcare provider, and (v) access electronic health records and medical notes.

**Results:**

41% of survey respondents identified as non-Hispanic Black individuals, 33% as non-Hispanic White individuals, and 22% as Hispanic individuals. 69% reported a pre-tax annual household income of less than $35,000. 97% reported ownership/access to a smart device; 97% reported access to reliable internet. In the past year, only 59% reported using the internet to better understand their medical diagnosis, 36% reported filling a prescription online, 47% scheduled a medical appointment online, 47% viewed electronic health records online, and 56% emailed healthcare providers. Female sex, higher incomes, and having at least a bachelor’s degree were significantly associated with all five online health information-seeking attributes.

**Conclusion:**

Despite high technology adoption rates, we observed suboptimal online health information-seeking behaviors. This underutilization has potential adverse implications for healthcare access and use given the documented advantage of HIT. Efforts to increase health information-seeking behaviors should explore the identification of HIT barriers, and patient education to increase familiarity and usage in this population.

## Introduction

In recent years, the digital revolution has significantly altered the healthcare landscape, transforming access to care, healthcare delivery, patient engagement, and the growth of online health information seeking behavior (HISB). With these purported benefits, digital access is being increasingly recognized as a social determinant of health (1). However, the “digital divide”, which refers to disparate access to information and communication technologies such as computers and the internet (2), may lead to greater health inequities (3). Multiple studies have shown a decreased likelihood of internet access and use for health information among historically underserved groups including minoritized race/ethnicity, limited English proficiency, lower education levels, older age, and lower-income households and rural residents (4, 5). A 2021 survey however showed evidence that the gap is narrowing rapidly, with 85% of White respondents, 83% of Black respondents, 85% of Hispanic respondents, and 76% of respondents making under $30,000 reporting smartphone ownership (6).

Despite increased ownership of smartphones and access to the internet in recent years, disparities in the use of digital technologies remain. This persistent disparity is worrisome because having the skills necessary to access credible online health information and the frequency of use has been associated with the perceived benefits of internet access (7). As information on health, healthcare, and social services are increasingly offered online, persistent internet inequity may disproportionately affect people with a greater need for these services and information. Considering the paucity of recent studies examining internet access and HISB among residents of low-income communities, this brief report examines access to technology, internet usage, and online health information-seeking behaviors, in a racially diverse, lower-income population.

## Methods

A cross-sectional survey was disseminated to adult residents (≥18 years) of low-income communities in Houston (Third Ward, East End), New York (Bronx, Brooklyn, Queens) and Los Angeles (East Los Angeles, Hyde Park, Huntington Park), between April 2023 and August 2023. These three geographically diverse metropolitan areas are represented in the top five largest metropolitan areas in the US, and were chosen as an attempt to make the sample more representative. To reach individuals who may not have access to technology, the research team worked with local community-based organizations to disseminate paper surveys in the target communities. The survey assessed access to technology, internet usage, and perceptions regarding health information obtained from the internet, in the target population. The survey included previously published questions, drawing from the Pew Research Center, the National Health Interview Survey, the Health Information National Trends Surveys, and other published work on this topic (8). Respondents spent on average 8 min to complete the survey. Binary responses to the following online health information-seeking behaviors, over the past 12 months, were examined: (i) internet use to better understand a medical diagnosis, (ii) internet use to fill a prescription, (iii) internet use to schedule an appointment with a healthcare provider, (iv) communicated with a healthcare provider by email, and (v) internet use to view electronic health records and medical notes. A total of 305 surveys were returned, of which 213 were complete (69.8% completion rate). Descriptive analyses employing frequencies and proportions were used to describe respondent characteristics. Chi-square tests were used to assess independent bivariate associations between survey respondent characteristics and the five aforementioned online health information seeking patterns.

## Results

**Figure 1:**
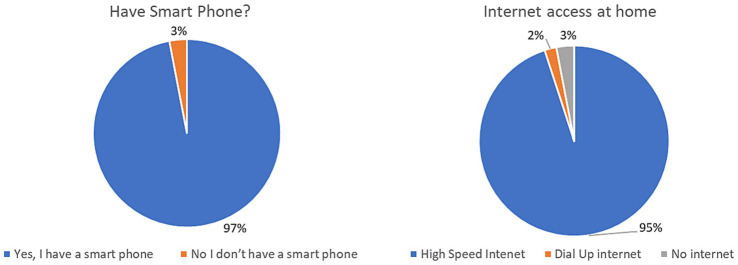
Access to smart phone and internet at home.

Table 1 shows the summary characteristics of the surveyed sample. Overall, 41% of survey respondents identified as non-Hispanic Black individuals, 33% as non-Hispanic White individuals, 22% as Hispanic individuals, and less than 4% identified as Middle Eastern/Asian/Native American/Pacific Islander/Other. About half of the respondents were aged 40–64 years, and over two-thirds (69%) reported a pre-tax annual household income of less than $35,000. Fifty-seven percent of respondents were female and only 21% reported having a bachelor’s degree or more. Ownership/access to a smart device was high, with over 97% indicating they owned a smartphone. Access to high-speed internet was also high, with over 95% responding affirmatively, and an additional 2% having access to dial-up internet (Figure 1).

**Table 1:** Bivariate associations: Respondents use of the internet for five (5) online health information seeking scenarios.

	Total sample	Used the internet to better understand a medical diagnosis.	*p*	Used the internet to fill a prescription	*p*	Used the internet to schedule an appointment with a health care provider	*p*	Communicated with a health care provider by email.	*p*	Used internet to view electronic health records and medical notes.	*p*
**Race/ethnicity**			0.08		0.84		0.03		0.13		0.00
White	71 (33.5)	38 (53.5)		25 (35.2)		29 (40.9)		34 (47.9)		21 (29.6)	
Black	86 (40.6)	58 (71.6)		33 (39.8)		47 (56.6)		52 (61.9)		50 (58.1)	
Hispanic	47 (22.2)	25 (53.2)		15 (31.9)		17 (36.2)		26 (55.3)		19 (40.4)	
Other	8 (3.7)	5 (62.5)		3 (37.5)		6 (75.0)		6 (85.7)		6 (75.0)	
**Age group**			0.15		0.26		0.63		0.64		0.24
18–39	59 (27.7)	30 (51.7)		17 (28.8)		28 (48.3)		30 (51.7)		27 (45.8)	
40–64	106 (49.8)	64 (62.1)		43 (41.4)		52 (50.0)		60 (57.7)		53 (50.0)	
65+	48 (22.5)	33 (70.2)		16 (34.0)		20 (41.7)		29 (60.4)		17 (35.4)	
**Income**			0.00		0.00		0.00		0.00		0.00
≤$35,000	146 (68.9)	75 (51.7)		44 (30.3)		53 (36.3)		71 (48.6)		46 (31.5)	
$35,001–$50,000	24 (11.3)	17 (70.8)		7 (29.2)		12 (52.2)		14 (58.3)		14 (58.3)	
$50,001–$75,000	16 (7.6)	12 (85.7)		8 (50.0)		13 (81.3)		11 (68.8)		12 (75.0)	
>$75,000	26 (12.2)	22 (91.7)		16 (66.7)		21 (87.5)		22 (95.7)		24 (92.3)	
**Education**			0.00		0.03		0.00		0.01		0.00
High School or less	93 (43.7)	51 (56.0)		34 (36.6)		34 (37.0)		44 (47.8)		27 (29.0)	
Technical/Vocational, Some College	76 (35.7)	38 (50.7)		20 (27.0)		34 (45.3)		43 (56.6)		36 (47.4)	
Bachelor’s degree or more	44 (20.6)	38 (90.5)		22 (51.2)		32 (74.4)		32 (76.2)		34 (77.3)	
**Sex**			0.01		0.04		0.03		0.01		0.02
Male	93 (43.7)	47 (51.1)		26 (28.3)		36 (39.1)		43 (46.7)		34 (36.6)	
Female	120 (56.3)	80 (69.0)		50 (42.4)		64 (54.2)		76 (64.4)		63 (52.5)	
Overall % responding “yes”		126 (59.2%)		76 (35.7%)		99 (46.5%)		118 (56.4%)		96 (46.54%)	

### Bivariate associations

Bivariate associations of independent variables by internet behaviors are also shown in Table 1. 59.2% of respondents reported using the internet to better understand their medical diagnosis. Those who used the internet to better understand their medical diagnosis were more likely to make over $75,000 per year (91.7% making >$75,000 vs. 85.7% making $50,001–$75,000, 70.8% making $35,001–$50,00, and 51.7% making ≤$35,000, *p* ≤ 0.01), hold a bachelor’s degree or more (90.5% bachelor’s degree or more vs. 50.7% technical/vocational or some college, and 56.0% with a high school degree or less, *p* ≤ 0.01) and be female (69.0% females vs. 51.1% males, *p* = 0.01).

Only 35.7% of respondents reported having used the internet to fill a prescription. However, respondents who filled prescriptions online were more likely to make over $75,000 per year (66.7% making >$75,000 vs. 50.0% making $50,001–$75,000, 29.2% making $35,001–$50,000, and 30.3% making ≤$35,000, *p* ≤ 0.01), hold a bachelor’s degree or more (51.2% bachelor’s degree or more vs. 27.0% technical/vocational or some college vs. 36.6% with a high school degree or less, *p* = 0.03), and be female (42.4% female vs. 28.3% males, *p* = 0.04).

Less than half of respondents (46.5%) reported using the internet to schedule an appointment with a health care provider. Those who scheduled appointments online were more likely to make over $75,000 per year (87.5% making >$75,000 vs. 81.3% making $50,001–$75,000 vs. 52.2% making $35,001–$50,000 vs. 36.3% making ≤$35,000, *p* ≤ 0.01), hold a bachelor’s degree or more (74.4% bachelor’s degree or more vs. 45.3% technical/vocational or some college, vs. 37.0% with a high school degree or less, *p* ≤ 0.01), and be female (54.2% vs. 39.1%, *p* = 0.03).

About half of the respondents (56.4%) reported using the internet to email a healthcare provider. Those who reported emailing providers in the past year were more likely to make over $75,000 per year (95.7% making >$75,000 vs. 68.8% making $50,001–$75,000, 58.3% making $35,001–$50,000, and 48.6% making ≤$35,000, *p* ≤ 0.01), hold a bachelor’s degree or more (76.2% bachelor’s degree or more vs. 56.6% technical/vocational or some college, and 47.8% high school degree or less, *p* = 0.01), and be female (64.4% females vs. 46.7% males, *p* = 0.01).

46.5% reported using the internet to view electronic health records and medical notes. Affirmative responses were significantly greater among other races (75.0% Other vs. 29.6% White, 58.1% Black, and 40.4% Hispanic, *p* ≤ 0.01), and among those who make over $75,000 per year (92.3% > $75,000 vs. 75.0% $50,001–$75,000, 58.3% making $35,001–$50,000, and 31.5% making ≤$35,000, *p* ≤ 0.01). Education was also strongly associated with using the internet to view electronic health, with respondents holding a bachelor’s degree or more being over-represented in this internet use attribute (77.3% bachelor’s degree or more vs. 47.4% technical/vocational or some college, and 29.0% high school degree or less, *p* ≤ 0.01). Females were more likely than males (52.5% females vs. 36.6% males, *p* = 0.02), to report using the internet to view electronic health records and medical notes.

## Discussion

This study examined online health-related seeking behaviors in racially diverse, lower-income communities and found that despite almost universal access to smart technology and the internet, there was an overall lukewarmness regarding the five online HISB. This aligns with general patterns reported by Vangeepuram et al. in their study of a low-income and racial/ethnic minority population, which found low health app utilization despite a high percentage of ownership (88% compared to our 97%) (9). The persistent, lower likelihood of engaging in HISB among people with lower education levels could point to the perceived utility of the internet for health-related matters or a gap in comprehension of digital health resources. Lower rates of online health information seeking may also be associated with lower perceived trust in online health communication channels and resources (10). This is concerning considering the ongoing proliferation of digital health technological modalities and highlights the need to explore and address barriers to HISB.

In our findings, using the internet to better understand one’s medical diagnosis was associated with female sex, higher income, and higher educational attainment. This behavior is important and has been associated with many benefits, including the potential for more productive health visits, accessibility, immediacy of information, and understanding of health concerns (11). Our finding is consistent with work by Alhusseini et al., who found that females, those with some college education, and those with a household income of at least $50,000 per year are more likely to seek online health information (12). Prior research on using the internet to better understand one’s medical diagnosis suggests that individuals seek a combination of information on illness and wellness (13). One study comparing predictors of HISB in 2002 and 2012 revealed that in both years, disease was the dominant online search topic, followed by treatment and healthy behaviors (14). Individuals with denser medical histories were also more likely to exhibit increased online HISB (14). Another study noted that the most common source of online health information was forums, and this behavior was most common among patients who gave lower patient-centered communication ratings to their providers, and/or experienced heightened worry due to the visit (15). Among males, lower income, and less educated respondents, a lower likelihood of using the internet to better understand one’s medical diagnosis could point to a lack of acute/chronic health issues and/or concerns about the credibility of sources (11).

Both filling a prescription and communicating with a health provider via email were associated with female sex, higher income, and higher educational attainment. Likewise, scheduling appointments with a healthcare provider and viewing electronic health records and medical notes online were both associated with race/ethnicity, female sex, higher income, and higher educational attainment.

This could be due to previously documented reasons such as difficulties with signing up and using patient portals to access electronic health records, especially for patients with limited health or digital literacy (16). However, work by Calixte noted that non-Hispanic Black individuals are less likely to fill prescriptions online compared to non-Hispanic Whites individuals (17). Similarly, an analysis by Ganeshan et al. found that minoritized patients, non-English speaking, and Medicaid recipients were less likely to schedule appointments online (18).

Surprisingly, age was not associated with any of the health information-seeking behaviors examined in this study. However, other studies link older age with lower odds of online health—information-seeking behaviors (14, 19). Work by Oh et al. on older adults’ usage of smartphones for HISB expands upon this, linking younger age, higher education levels, regular exercise, higher medical expenses, and higher health literacy as predictors of HISB (20). This difference may be reflected in lower e-health literacy and concerns about using the Internet to diagnose or treat health issues. A study by Silver et al. on patients aged 50 years and older aggregated concerns into six high-level categories: limitations in own ability (fear of misdiagnosing or making things worse), credibility/limitations of online information, anxiety (feeling overwhelmed or worse after looking online), time consumption, conflict (fears of upsetting doctor or family), and non-physical harm (fears of getting scammed or having information stolen) (21). Despite these concerns, 48% of the older adult participants reported they would feel comfortable recording their health information online (21). Age-related underutilization could be indicative of distrust towards the use of new technologies but importantly presents an opportunity to address this gap through multisectoral approaches.

Our study does have limitations that should be considered when interpreting the results. To accommodate the needs of our community partners, we created a new survey tool that drew on multiple sources, rather than using a single previously validated tool such as the eHealth Literacy Scale (eHEALS) (22) or the HLS19-DIGI Instrument to measure Digital Health Literacy (23). Also, we were not able to collect participant reasons for non-completion of the survey tool, so there is a possibility of non-response bias reflected in our results.

In conclusion, the equitable distribution of health technology benefits among all strata of society needs more attention. Bridging the digital divide in healthcare and accommodating vulnerable populations, particularly racially diverse and lower-income populations, is crucial to promoting equitable healthcare access, treatment, and outcomes.

## Data availability statement

The datasets used in this study are protected as part of the IRB approval. Further inquires should be directed to the corresponding author.

## Ethics statement

The studies involving humans were approved by University of Houston Institutional Review Board. The studies were conducted in accordance with the local legislation and institutional requirements. The ethics committee/institutional review board waived the requirement of written informed consent for participation from the participants or the participants’ legal guardians/next of kin because language about consent was included on the first page of the survey, during the recruitment process when participants indicated a willingness to engage.

## Author contributions

OA: Conceptualization, Data curation, Investigation, Methodology, Software, Supervision, Validation, Writing – original draft, Writing – review & editing. MS: Writing – original draft. MTi: Writing – review & editing, Data curation, Formal analysis. GP: Project administration, Writing – review & editing. MTr: Conceptualization, Project administration, Writing – review & editing. CO: Methodology, Writing – review & editing.
